# 760. Incidence, Predictors and 30-Day Outcomes of *Clostridoides difficile* Infection in Patients Undergoing Cystectomy: A Nationwide Analysis Using the ACS-NSQIP Database

**DOI:** 10.1093/ofid/ofab466.957

**Published:** 2021-12-04

**Authors:** Armaghan-e-Rehman Mansoor, Yousaf B Hadi, Mohamad Salkini, Arif R Sarwari

**Affiliations:** 1 Washington University in St. Louis, Brentwood, Missouri; 2 West Virginia University, Morgantown, West Virginia

## Abstract

**Background:**

Clostridoides difficile infection is the second most common healthcare acquired infection (HAI) and the most common gastrointestinal HAI, with an estimated 365,200 cases reported by the CDC in 2017. CDI continues to remain a major cause of inpatient admission and utilization of healthcare resources. The exact incidence of peri-procedural CDI with cystectomy is unknown, and reported incidence of CDI in literature vary widely

**Methods:**

We conducted an analysis of patients undergoing cystectomy between 2015 and 2017 using the ACS National Surgical Quality Improvement Program (NSQIP) to study the incidence, risk factors and 30-day post-surgical outcomes associated with CDI following cystectomy. Developed by the American College of Surgery, this is a nationally validated, risk-adjusted, outcomes-based program designed to determine and improve the quality of surgical and post-surgical care.

**Results:**

The incidence of CDI following cystectomy was 3.6% in our patient cohort. 18.8% of patients developed CDI following hospital discharge. Non-elective surgeries, and complete cystectomy procedures had higher rate of CDI. 48.4% of patients with CDI had a preceding post-operative infection. Post-operative organ space infections (OR 1.95), post-operative renal failure (OR 2.38), post-operative sepsis (OR 2.49) and septic shock (OR 2.33) were independently associated with development of CDI, (all p values < 0.05). Patients who developed post-operative CDI during hospitalization had lengthier hospital admissions than those who did not develop a CDI (OR 2.29) and had a higher risk of DVT formation (2.48), and were also more likely to have unplanned readmissions (OR 7.8)

**Conclusion:**

This is the first nationwide study looking at inpatient and 30-day post-operative CDI after cystectomy in the US. A sizable number of patients experience CDIs after cystectomy procedures, and CDI development is associated with an increase in length of stay and unplanned readmissions. This study lends further evidence to the need for continued interventions and initiatives to reduce this burden of post-operative CDI.

**Disclosures:**

**All Authors**: No reported disclosures

